# Differential Sphingosine-1-Phosphate Receptor-1 Protein Expression in the Dorsolateral Prefrontal Cortex Between Schizophrenia Type 1 and Type 2

**DOI:** 10.3389/fpsyt.2022.827981

**Published:** 2022-03-08

**Authors:** Ganesh B. Chand, Hao Jiang, J. Philip Miller, C. Harker Rhodes, Zhude Tu, Dean Foster Wong

**Affiliations:** ^1^Mallinckrodt Institute of Radiology, Washington University School of Medicine, St. Louis, MO, United States; ^2^Division of Biostatistics, Washington University School of Medicine, St. Louis, MO, United States; ^3^NeuroDex Inc., Natick, MA, United States; ^4^Department of Psychiatry, Neuroscience, and Neurology, Washington University School of Medicine, St. Louis, MO, United States

**Keywords:** autoradiography, schizophrenia, sphingosine-1-phosphate receptor-1 (S1PR1), postmortem brain tissues, molecular imaging, neuroimaging

## Abstract

Understanding the etiology and treatment approaches in schizophrenia is challenged in part by the heterogeneity of this disorder. One encouraging progress is the growing evidence that there are subtypes of schizophrenia. Recent *in vitro* findings of messenger ribonucleic acid (mRNA) gene expression on postmortem dorsolateral prefrontal cortex (DLPFC) showed that schizophrenia has two subtypes, those with a relatively normal DLPFC transcriptome (Type 1) and those with differentially expressed genes (Type 2). Sphingosine-1-phosphate receptor-1 (S1PR1) is one of the genes that was highly upregulated in Type 2 compared to Type 1 and controls. The impact of that finding is limited because it only can be confirmed through analysis of autopsy tissue, and the clinical characteristics such as symptoms severity or illness duration except for cause of death was not available from that Medical Examiner based autopsy study. However, S1PR1 has great potential because it is a target gene that can be accessed *via* positron emission tomography (PET) *in vivo* using specific radioligands (starting with [^11^C]CS1P1) successfully developed at our center in human brain imaging. As a preliminary study to validate this PET target in schizophrenia, S1PR1 protein expression was assessed by receptor autoradiography (ARG) using [^3^H]CS1P1 and immunohistochemistry (IHC) in the DLPFC from patients with schizophrenia classified as Type 1 or Type 2 based on their DLPFC transcriptomes and from controls. Our analyses demonstrate that ARG S1PR1 protein expression is significantly higher in Type 2 compared to Type 1 (*p* < 0.05) and controls (*p* < 0.05), which was consistent with previous mRNA S1PR1. These findings support the possibility that PET S1PR1 can be used as a future imaging biomarker to distinguish these subgroups of schizophrenic patients during life with obvious implications for both patient management and the design of clinical trials to validate novel pharmacologic therapies.

## Introduction

Schizophrenia is a neuropsychiatric condition that currently affects ~3 million people in the United States and ~7.8 billion people worldwide (1–3). Individuals with schizophrenia exhibit highly heterogeneous genetic profiles (4), clinical symptoms (5), illness course (6, 7), treatment response (8, 9), and neuroimaging markers (10–12). Despite extensive efforts, understanding schizophrenia mechanisms remains challenging. Even though typically in mRNA studies one generally looks at gene expressions in context and examine a large number of dysregulated mRNAs that are involved in mitochondrial and proteasome functions, the direct link between these functions and schizophrenia mechanisms/features remain unclear (13, 14). While decreases in Cannabinoid (CB) mRNA that targets GABA interneurons were previously reported in schizophrenia (15), OMAR CB1 PET radioligand showed elevations in the same schizophrenia tissues (16) suggesting that this strategy is not as fruitful in developing schizophrenia treatment. Recent findings by Bowen et al. (17) suggest that the mRNA expression can be used to divide schizophrenic patients into two types, Type 1 schizophrenia patients with an essentially normal transcriptome in their dorsolateral prefrontal cortex (DLPFC) and Type 2 schizophrenia patients with hundreds of differentially expressed genes in their DLPFC. Although there are many highly upregulated interesting genes including S1PR1 (*p* < 10^−15^; after multiple comparisons) in Type 2 (17), S1PR1 is only one that has currently been developed and the radioligands for the other target genes are not available yet. Additionally, S1PR1 radioligand has gained significant interest for *in vivo* targeted imaging of inflammation in brain diseases, with the recent FDA-approved S1PR1-based treatments such as Fingolimod, Siponimod, and Ozanimod (18) for multiple sclerosis.

However, a serious limitation of mRNA expression studies like that of Bowen et al. (17) is that they require brain tissue which is generally not available except at autopsy. Fortunately, PET ligands for S1PR1 have been recently developed at Washington University School of Medicine (19–24). The present study examines the differential expression of S1PR1 in the DLPFC of Type 1 and Type 2 schizophrenic patients at the protein level as a preliminary step toward the use of PET to distinguish Type 1 from Type 2 schizophrenia during life. We performed ARG and IHC analyses in DLPFC tissues of controls, Type 1 and Type 2 schizophrenic patients. Since ARG is a more accurate quantitative method compared to IHC (25, 26), ARG was used for S1PR1 quantitation while IHC was used only to confirm ARG signals. We hypothesized that S1PR1 protein expression will show elevations in Type 2 schizophrenia compared to Type 1 schizophrenia and controls consistent with Bowen's S1PR1 mRNA findings (17).

## Materials and Methods

### Human Brain Tissues

Human brain tissues were obtained from the Human Brain Collection Core at the National Institute of Mental Health. These tissues corresponded to the same tissues studied by Bowen et al. (17) because any other tissues from different sources would be difficult to distinguish Type 1 and Type 2 schizophrenia. Tissues were used in accordance with the guidelines of Washington University in St. Louis. All samples were stored at −80°C at the Washington University's Radiology labs until used.

### *In vitro* Immunohistochemistry Staining Study

*In vitro* IHC staining of S1PR1 was carried out in frozen sections from human DLPFC. All sections were pre-warmed at room temperature (RT) for 5 min and then fixed with 4% paraformaldehyde in phosphate buffered saline (PBS) for 10 min at RT, washed 3 times in PBS, and then heated in boiling water bath in antigen retrieval buffer for 30 min. Sections were then rinsed with PBS and blocked with 5% horse serum for 1 h at RT. After that, all sections were stained with anti-S1PR1 antibody (Alomone, Jerusalem, Israel) overnight at 4°C, washed and followed by incubation with ImmPRESS HRP Horse anti-rabbit polymer for 1 h at RT, and developed using ImmPACT DAB (Vector Laboratories, Burlingame, CA). Hematoxylin and eosin (H&E) staining was also performed in adjacent slides to identify gray and white matters in the brain.

### *In vitro* Autoradiography Study

*In vitro* ARG study was carried out in frozen sections from human DLPFC using [^3^H]CS1P1 for S1PR1 receptor protein. Sections were pre-warmed to RT, and then incubated with Hank's balanced salt solution (HBSS) buffer containing 10 mM HEPES, 5 mM MgCl_2_, 0.2% BSA, and 0.1 mM EDTA at pH 7.4 for 5 min at RT in a coplin jar. All sections were then incubated with 0.5 nM [^3^H]CS1P1 for 30 min in a coplin jar with gentle shaking at RT. After that, all sections were washed with a buffer for 3 min for three times, and then rinsed in ice-cold H_2_O for 1 min and air dried overnight. Slides were incubated with Carestream BioMax Maximum Sensitivity ARG film (Carestream, Rochester, NY) in a Hypercassette ARG cassette (Cytiva, Amersham, UK) for 30 days along with an ART-123 Tritium Standards (American Radiolabeled Chemicals, St Louis, MO). The film was processed using a Kodak film developer (Kodak, Rochester, NY). To determine the non-specific binding, 10 μM of S1PR1 specific antagonist NIBR-0213 (Cayman, Ann Arbor, MI) was introduced and incubated with samples as described above. The image was processed and analyzed using Fiji ImageJ. Brain regions of interest (ROIs) were selected from the ARG images according to the hematoxylin staining in the adjacent slide. ROIs were randomly selected from different regions of the DLPFC gray matter, and the intensity was measured and calculated in fmol/mg.

### Statistical Analysis

In statistical analysis, we fitted a mixed repeated measure model to take into account the triplicate measure variability as well as the interpersonal variability (within group). In mixed model analysis of variance (ANOVA), the groups were used as a fixed effect. The *F*-test for the null that all three groups have the same mean was tested. To compare between groups, a one-tailed *t*-test was used. A *p* ≤ 0.05 was considered statistically significant. Statistical calculations were performed with PROC MIXED in SAS 9.4.

## Results

### Postmortem Human Subjects

DLPFC tissues from 20 human subjects including 10 neurologically normal controls, five Type 1 schizophrenia subjects, and five Type 2 schizophrenia subjects were used in this study (Table 1). Mean age (standard deviation) was 55.20 years (9.69) of normal controls, 56.40 years (8.32) of Type 1 schizophrenia, and 53.20 years (10.26) of Type 2 schizophrenia subjects. There was one female in controls, none in Type 1 schizophrenia, and one in Type 2 schizophrenia. There were five Caucasians and five African-Americans in controls, three Caucasians and two African-Americans in Type 1 schizophrenia, and two Caucasians and three African-Americans in Type 2 schizophrenia. Age, sex, and race were not significantly different among groups. Each subject's cause of death and manner of death are also included in the footnote of Table 1.

**Table 1:** Human DLPFC tissues used in this study from normal controls, schizophrenia Type 1 and Type 2 patients.

**Normal**				**Schizophrenia Type 1**				**Schizophrenia Type 2**			
**Subject**	**Sex**	**Race**	**Age**	**Subject**	**Sex**	**Race**	**Age**	**Subject**	**Sex**	**Race**	**Age**
1	M	CAUC	54	1	M	AA	60	1	F	AA	63
2	F	AA	64	2	M	AA	53	2	M	CAUC	48
3	M	AA	57	3	M	CAUC	59	3	M	AA	53
4	M	AA	64	4	M	CAUC	66	4	M	AA	63
5	M	CAUC	49	5	M	CAUC	44	5	M	CAUC	39
6	M	CAUC	51								
7	M	AA	58								
8	M	CAUC	32								
9	M	AA	60								
10	M	CAUC	63								

*F, Female; M, Male; AA, African American; CAUC, Caucasian; Age in years*.

***Normal subjects: Cause of death (Manner of death)***.

*Subject 1: Hypertensive and arteriosclerotic cardiovascular disease (Natural)*.

*Subject 2: Cardiac tamponade due to acute dissecting aneurysm of aortic arch due to atherosclerotic hypertensive cardiovascular disease (Natural)*.

*Subject 3: Heat exposure associated with pulmonary emphysema (Accident)*.

*Subject 4: Acute pulmonary thromboembolism due to deep venous thrombosis due to immobility following right inguinal herniorrhaphy (Natural)*.

*Subject 5: Cardiomyopathy (Natural)*.

*Subject 6: Acute myocardial infarction due to atherosclerotic cardiovascular disease (Natural)*.

*Subject 7: Poorly differentiated pulmonary adenocarcinoma (Natural)*.

*Subject 8: Cardiac arrest of undetermined etiology during repair of paraesophageal hernia (Natural)*.

*Subject 9: Acute myocardial infarction, secondary to severe atherosclerotic heart disease (Natural)*.

*Subject 10: Massive spinal cord injury (Accident)*.

***Schizophrenia Type 1 subjects: Cause of death (Manner of death)***.

*Subject 1: Hypertensive and atherosclerotic cardiovascular disease (Natural)*.

*Subject 2: Non-small cell carcinoma of lung with erosion of primary branch of pulmonary artery (Natural)*.

*Subject 3: Multiple blunt force injuries (Suicide)*.

*Subject 4: Atherosclerotic cardiovascular disease (Natural)*.

*Subject 5: Myocardial infarction (Natural)*.

***Schizophrenia Type 2 subjects: Cause of death (Manner of death)***.

*Subject 1: Hypertensive cardiovascular disease (Natural)*.

*Subject 2: Drowning (Accident)*.

*Subject 3: Acute bronchopneumonia due to hypertensive and atherosclerotic cardiovascular disease (Natural)*.

*Subject 4: Atherosclerotic hypertensive cardiovascular disease (Natural)*.

*Subject 5: Combined drug poisoning (clozapine and sertraline) (Accident)*.

### Immunostaining of S1PR1

**Figure 1:**
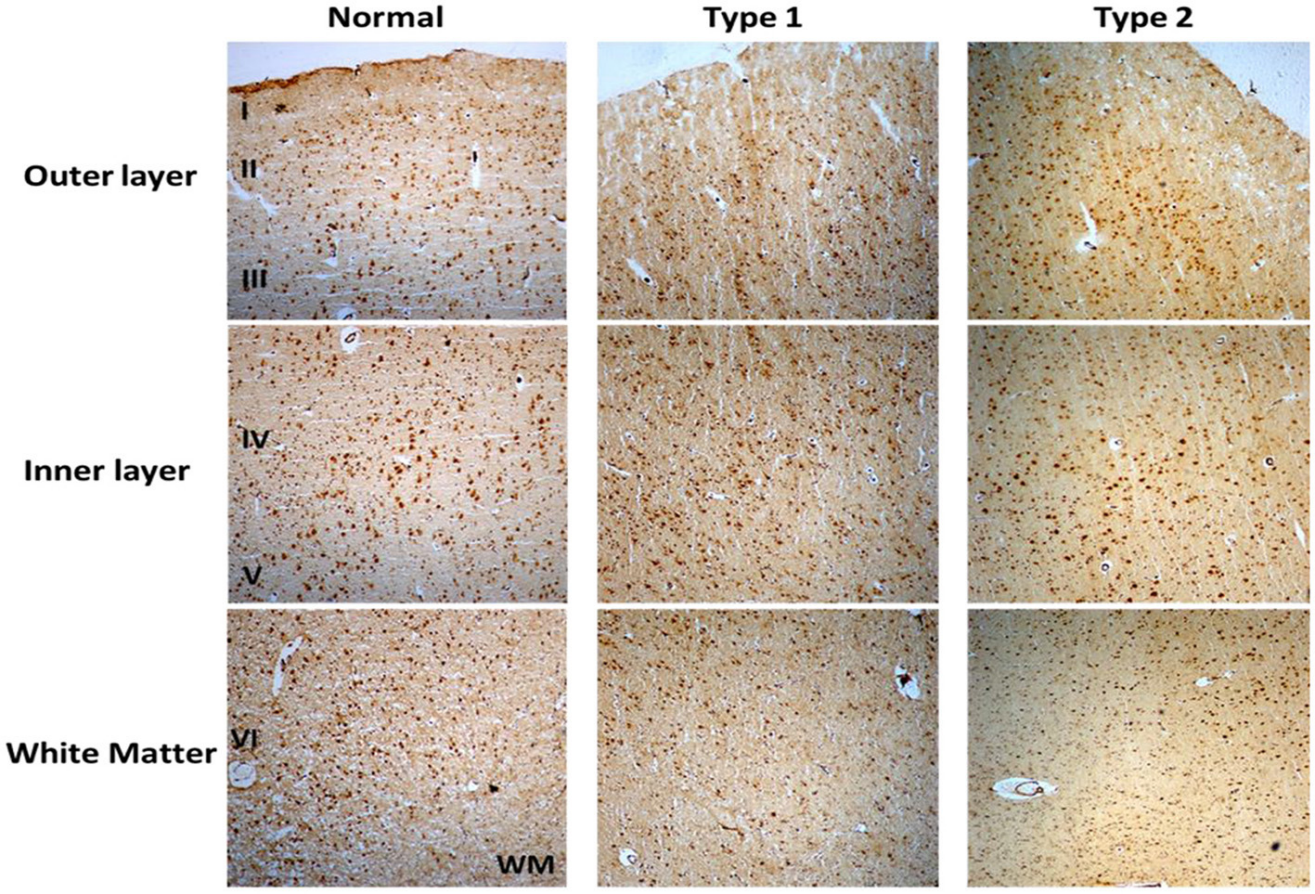
Immunohistochemistry (IHC) of S1PR1 in postmortem DLPFC tissues from the representative normal control and schizophrenia Type 1 and Type 2.

**Figure 2:**
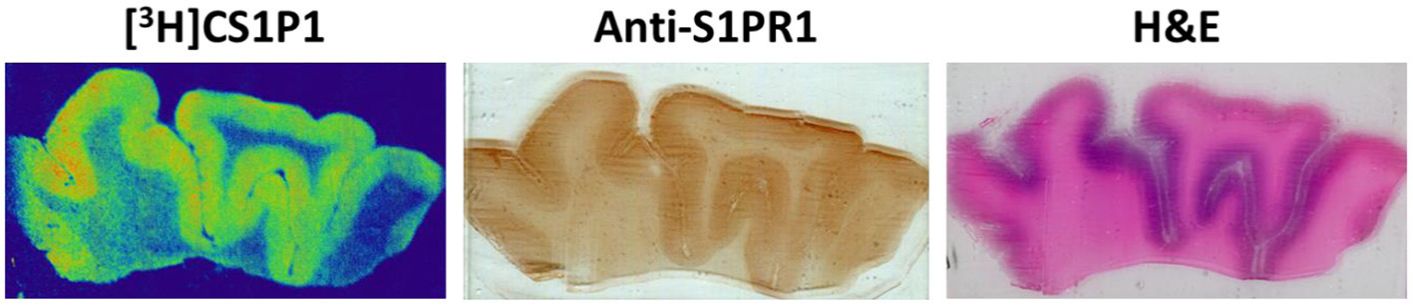
Representative images of [^3^H]CS1P1 autoradiograph, S1PR1 immunostaining, and Hematoxylin and eosin (H&E) staining in postmortem human DLPFC tissues. The distribution of [^3^H]CS1P1 matched well with anti-S1PR1 antibody, and was mainly located in the gray matter regions as indicated in the H&E staining.

Immunostaining of S1PR1 was performed in control and schizophrenia samples (Figures 1, 2). In general, S1PR1 was mainly expressed in the gray matter of the DLPFC. In particular, the expression of S1PR1 was relatively high in the outer granular layer, outer pyramidal layer, inner granular layer, inner pyramidal layer, and multiform layer with very low to no amount in the molecular layer of gray matter and white matter.

### Autoradiography of S1PR1 Specific [^3^H]CS1P1

ARG analysis of S1PR1 was performed in control and schizophrenia DLPFC samples.

**Figure 3:**
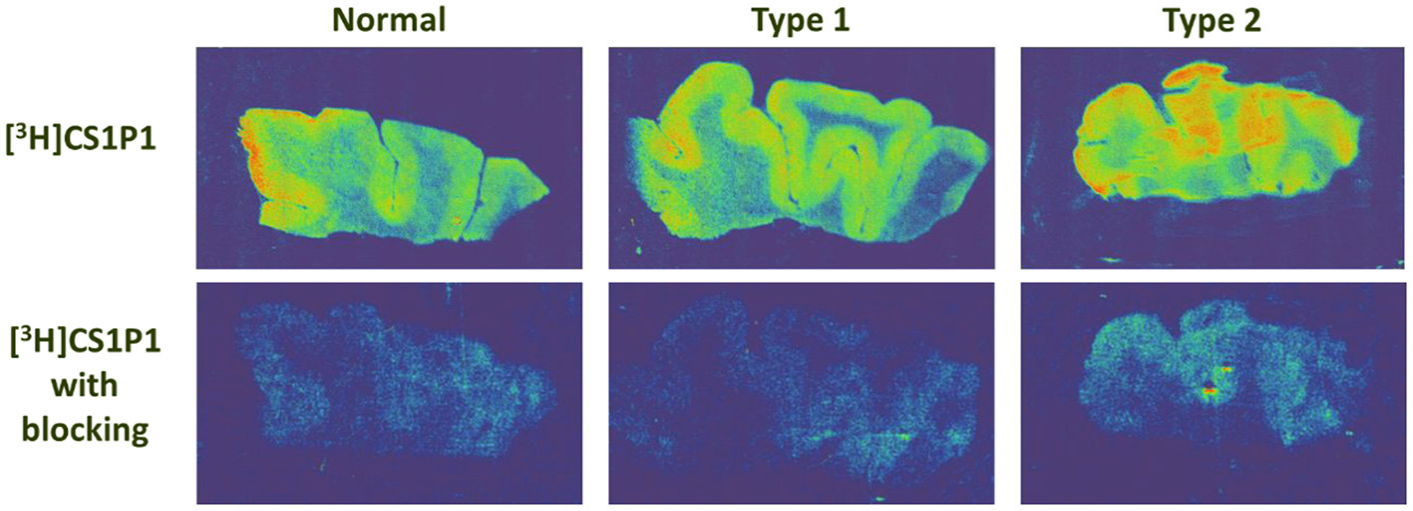
Autoradiography images of S1PR1 using [^3^H]CS1P1 in postmortem DLPFC tissues from representative normal control, schizophrenia Type 1, and schizophrenia Type 2. In general, [^3^H]CS1P1 was higher in Type 2 schizophrenia subjects compared with normal control and Type 1 schizophrenia subjects.

The distribution pattern of [^3^H]CS1P1 matched well with immunostaining analysis using S1PR1 specific antibody, indicating [^3^H]CS1P1 is specific to S1PR1 in postmortem human tissues (Figures 2, 3). Similar to S1PR1 immunostaining analysis, S1PR1 specific [^3^H]CS1P1 was mainly distributed in the gray matter of DLPFC, with no to very low amount of [^3^H]CS1P1 distributed in the white matter region. In addition, blocking study using S1PR1 antagonist NIBR-0213 showed significant reduction of [^3^H]CS1P1 indicating the [^3^H]CS1P1 is specific to S1PR1 (Figure 3). Compared with IHC analysis, ARG provides both quantification and localization of radioligand at the same time in distinct anatomical structures, and enables us to quantify the absolute amount of [^3^H]CS1P1 in control and different types of schizophrenia subjects.

ARG S1PR1 intensity in the DLPFC was compared between the groups. In general, the intensity of [^3^H]CS1P1 was higher in Type 2 schizophrenia subjects compared with normal control and Type 1 schizophrenia subjects (Figure 3).

**Figure 4:**
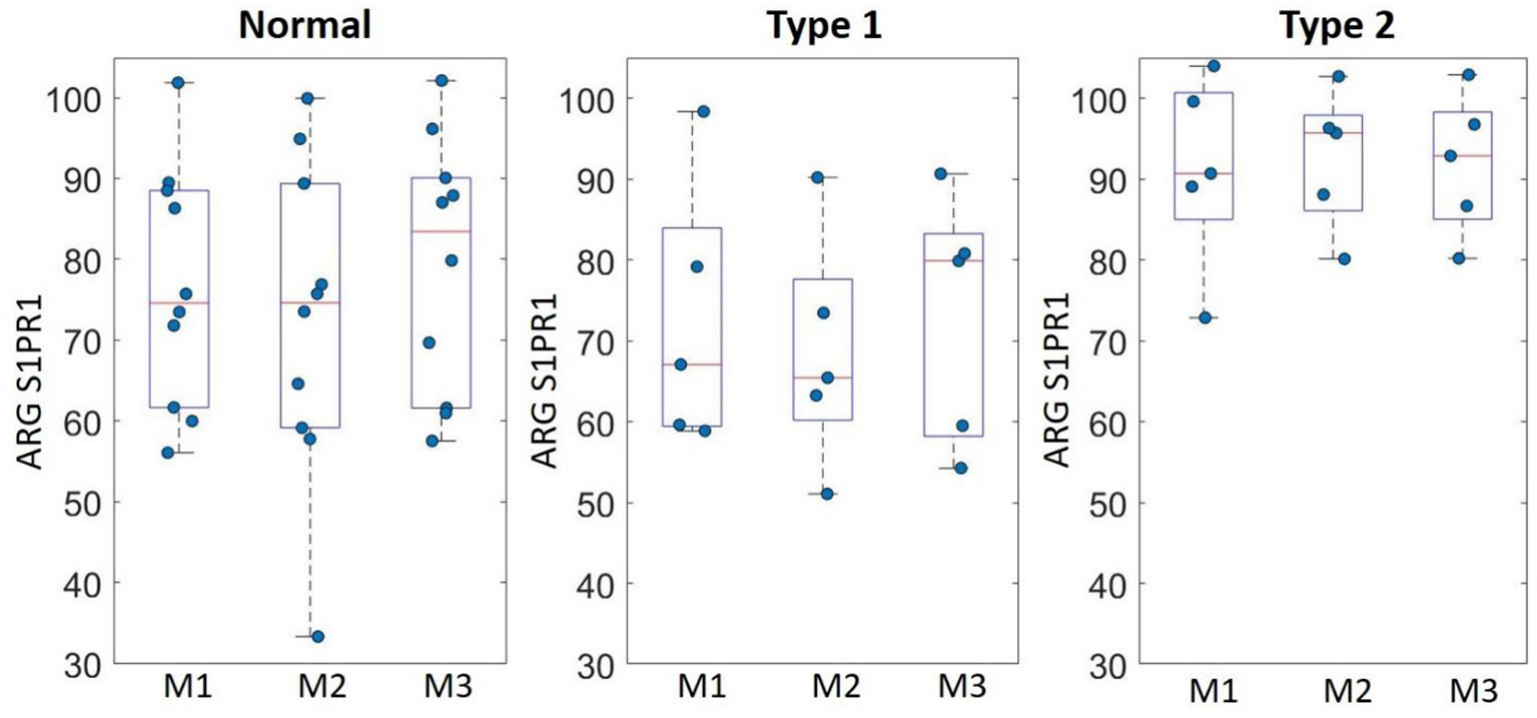
ARG S1PR1 intensity expression (fmol/mg) triplicate measures (M1, M2, and M3) in the DLPFC from normal controls, schizophrenia Type 1, and schizophrenia Type 2.

**Figure 5:**
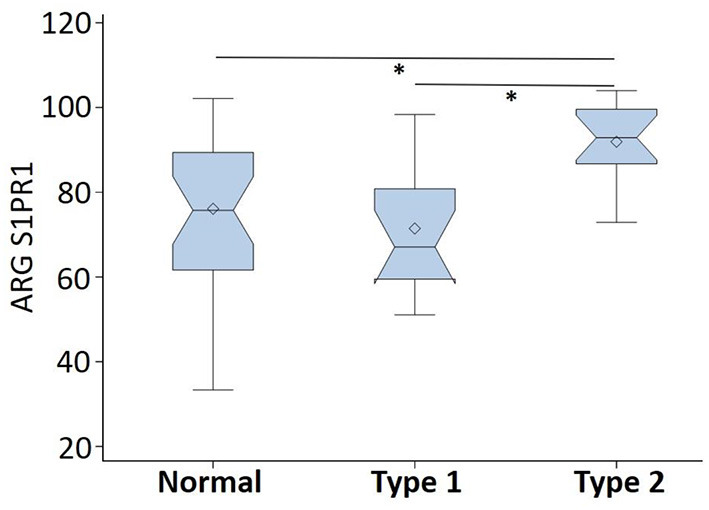
ARG S1PR1 intensity expression (in fmol/mg) comparison between normal controls, schizophrenia Type 1, and schizophrenia Type 2 (*p < 0.05).

ARG S1PR1 intensity expressions were measured at three randomly selected ROIs within the DLPFC in all subjects. These ARG S1PR1 triplicate measures from normal controls, Type 1 schizophrenia, and Type 2 schizophrenia are shown in Figure 4. AGR S1PR1 is highly expressed in Type 2 schizophrenia in all three measures compared to Type 1 schizophrenia and controls. The *F*-test for the null that all three groups have the same mean was *F*_(2, 17)_ = 3.49, *p* = 0.05. Overall ARG S1PR1 intensity mean (standard error) was 76.10 (4.66) for controls, 71.44 (5.87) for Type 1 schizophrenia, and 91.91 (5.87) for Type 2 schizophrenia (Figure 5). ARG S1PR1 expression was significantly higher in Type 2 schizophrenia compared to controls (t = 2.20, *p* = 0.021) and Type 1 schizophrenia (t = 2.47, *p* = 0.012), but there was no difference between controls and Type 1 schizophrenia (t = 0.65, *p* = 0.525).

## Discussion

In this study, we evaluated the expression of S1PR1 and distribution of S1PR1 specific tracer [^3^H]CS1P1 in human DLPFC tissues from normal control, Type 1 and Type 2 schizophrenia subjects. Our data showed, in the DLPFC of humans, S1PR1 is highly expressed in the gray matter region with much lower expression in the white matter regions. Similar to the immunostaining study, ARG analysis using [^3^H]CS1P1 showed a relatively high tracer uptake in the gray matter of DLPFC whereas no to very low amount of [^3^H]CS1P1 was identified in the white matter region. Tracer uptake in the Type 2 schizophrenia samples was significantly higher than the Type 1 schizophrenia samples and normal controls.

The present ARG findings are consistent with a previous study in other regions of the human brain (27) that suggests S1PR1 is mainly localized in gray matter and confirmed the specificity of our S1PR1 specific radioligand [^3^H]CS1P1. This study extends the previous findings (17) by demonstrating that S1PR1 protein as well as mRNA is differentially expressed in the DLPFC of Type 2 schizophrenic patients. The trend of tracer uptake between control and all schizophrenia subjects was similar to previous findings using RT-PCR (28). For the two types of schizophrenia patients, it appeared that only Type 2 schizophrenia has significantly upregulated S1PR1 compared to Type 1 schizophrenia and controls, respectively. Thus, the present ARG findings and previous reports taken together indicate that both S1PR1 protein and S1PR1 mRNA upregulate in DLPFC only in a subset of schizophrenia.

S1PR1 is localized in astrocytes (27), the most abundant glial cells in the brain. Prior study from our group (19) shows S1PR1 localization in astrocytes (interlaminar) and microglia. S1PR1 involvements have been suggested in astrocyte morphogenesis and in bi-directional communications between astrocytes and neurons (29). Our results using both immunostaining and ARG indicated that S1PR1 is highly expressed in the gray matter compared to white matter, which was consistent with previous studies in other brain regions (27). Since frozen DLPFC tissues were used, the immunostaining-based morphology will not be well-suited compared to fixed tissues. Present immunostaining results indicate S1PR1 looks like in astrocytes, but fixed tissues will need to be studied in future for S1PR1 cellular localization. Both IHC and ARG confirmed S1PR1 signals at macro level, but only ARG was used for S1PR1 quantitation as suggested previously (25, 26). The present results will be important for the future schizophrenia subtype-based studies with *in vivo* PET [^11^C]CS1P1. The [^11^C]CS1P1 has recently emerged as a promising radiotracer for *in vivo* PET imaging of neuro-inflammation (19, 21–23). Neuro-inflammation has been associated with schizophrenia throughout the literature (30, 31). This has included elevated cytokines (32, 33) that may be due to activated microglia. The neuro-inflammatory observation seems to be most associated with early illness stages and patients with acute psychosis symptom exacerbations (34). S1PR1 PET will potentially complement for investigating neuro-inflammation in schizophrenia with other currently used ligands, such as Translocator protein (18 kDa) (TSPO) PET (35–38). Future studies will be needed for the direct comparison of S1PR1 and TSPO. Previous literature and our present findings taken together thus suggest that there is great potential for studying neuro-inflammation (or related mechanisms) in schizophrenia with *in vivo* S1PR1 PET. If S1PR1 PET reveals biologically distinct subtypes of schizophrenia, the clinical relevance is that PET study can be used to stratify patient cohorts in therapeutic trials of new drugs. The implications for neurogenetic and epidemiologic studies are equally obvious. The only limitation is the cost of PET, especially for bigger sample studies. Modulators targeting S1PR1 are already FDA-approved therapeutics of treating multiple sclerosis (18, 39), which could further accelerate its important role for treatment development in schizophrenia.

### Limitations

One limitation is that present study as well as previous S1PR1 mRNA study (17) cannot be used to identify schizophrenia Type 1 and Type 2 *in vivo* without PET and S1PR1 specific radiotracer. However, there is mounting evidence that schizophrenia has two neuroanatomical types *in vivo* (40). This recent study by Chand et al. (40) can potentially be used to divide schizophrenia patients into types *in vivo* using structural MRI. To evaluate whether volumetric MRI-based schizophrenia subtypes (40) and S1PR1-based schizophrenia subtypes map each other, the combined *in vivo* MRI and S1PR1 PET studies will be needed in future. We also acknowledge that the sample size is relatively small for this study, and the future studies should focus on replicating these findings in larger samples. However, we were limited by the available tissues in the HBCC NIMH brain bank that corresponded to the same tissues studied by Bowen et al. (17), and any other tissues would be difficult to distinguish Type 1 and Type 2 schizophrenia. Another limitation of our study is that clinical characteristics such as symptoms severity and illness duration were not available and the relationships between these variables and subtypes remain unknown. Lastly, it remains to be investigated S1PR1 protein and S1PR1 mRNA expressions in other brain regions besides DLPFC of controls and Type 1 and Type 2 schizophrenia patients.

### Conclusions

The present study evaluated DLPFC postmortem tissues from controls, schizophrenia Type 1 and Type 2, demonstrated S1PR1 protein is highly expressed in gray matter region, and most importantly showed that only Type 2 schizophrenia has upregulated S1PR1 protein expression in line with previous S1PR1 mRNA results. Overall, these findings strongly suggest S1PR1 might serve as a candidate target in schizophrenia subtypes with PET where protein is the target.

## Data Availability Statement

Human brain tissue data used in this study are publicly available from the Human Brain Collection Core (HBCC) at the National Institute of Mental Health (NIMH) following the data request procedure. The original data presented in the study are included in the article, and further inquiries can be directed to the corresponding author (GC).

## Ethics Statement

The studies involving human participants were reviewed and approved by Human Brain Collection Core (HBCC) at the National Institute of Mental Health (NIMH). The patients/participants provided their written informed consent to participate in this study.

## Author Contributions

GC, CR, and DW conceived the project. HJ and ZT performed autoradiography and immunohistochemistry experiments. JM and GC performed data analyses. GC wrote the initial manuscript draft. GC, HJ, JM, CR, ZT, and DW critically reviewed and revised the manuscript draft, and approved the final version. All authors contributed to the article and approved the submitted version.

## Conflict of Interest

CR is employed by Neurodex Inc. The remaining authors declare that the research was conducted in the absence of any commercial or financial relationships that could be construed as a potential conflict of interest.

## Publisher's Note

All claims expressed in this article are solely those of the authors and do not necessarily represent those of their affiliated organizations, or those of the publisher, the editors and the reviewers. Any product that may be evaluated in this article, or claim that may be made by its manufacturer, is not guaranteed or endorsed by the publisher.

## References

[1] Chong HY Teoh SL Wu DBC Kotirum S Chiou CF Chaiyakunapruk N. Global economic burden of schizophrenia: a systematic review. Neuropsychiatr Dis Treat. (2016) 12:357–73. 10.2147/NDT.S96649 26937191 PMC4762470

[2] Cloutier M Aigbogun MS Guerin A Nitulescu R Ramanakumar AV Kamat SA. The economic burden of schizophrenia in the United States in 2013. J Clin Psychiatry. (2016) 77:764–71. 10.4088/JCP.15m10278 27135986

[3] Mccutcheon RA Reis Marques T Howes OD. Schizophrenia-an overview. JAMA Psychiatry. (2020) 77:201–10. 10.1001/jamapsychiatry.2019.3360 31664453

[4] Arnedo J Svrakic DM Del Val C Romero-Zaliz R Hernandez-Cuervo H Molecular Molecular Genetics of Schizophrenia C . Uncovering the hidden risk architecture of the schizophrenias: confirmation in three independent genome-wide association studies. Am J Psychiatry. (2015) 172:139–53. 10.1176/appi.ajp.2014.14040435 25219520 PMC12884332

[5] Derks EM Allardyce J Boks MP Vermunt JK Hijman R Ophoff RA. Kraepelin was right: a latent class analysis of symptom dimensions in patients and controls. Schizophr Bull. (2012) 38:495–505. 10.1093/schbul/sbq103 20864620 PMC3329975

[6] Carpenter WT Kirkpatrick B. The heterogeneity of the long-term course of schizophrenia. Schizophr Bull. (1988) 14:645–52. 10.1093/schbul/14.4.645 3064288

[7] Huber G. The heterogeneous course of schizophrenia. Schizophr Res. (1997) 28:177–85. 10.1016/S0920-9964(97)00113-8 9468352

[8] Palaniyappan L Marques TR Taylor H Handley R Mondelli V Bonaccorso S. Cortical folding defects as markers of poor treatment response in first-episode psychosis. JAMA Psychiatry. (2013) 70:1031–40. 10.1001/jamapsychiatry.2013.203 23945954 PMC7617342

[9] Malhotra AK. Dissecting the heterogeneity of treatment response in first-episode schizophrenia. Schizophr Bull. (2015) 41:1224–6. 10.1093/schbul/sbv117 26333841 PMC4601718

[10] Voineskos AN Foussias G Lerch J Felsky D Remington G Rajji TK. Neuroimaging evidence for the deficit subtype of schizophrenia. JAMA Psychiatry. (2013) 70:472–80. 10.1001/jamapsychiatry.2013.786 23467781

[11] Nenadic I Yotter RA Sauer H Gaser C. Patterns of cortical thinning in different subgroups of schizophrenia. Br J Psychiatry. (2015) 206:479–83. 10.1192/bjp.bp.114.148510 25657354

[12] Voineskos AN Jacobs GR Ameis SH. Neuroimaging heterogeneity in psychosis: neurobiological underpinnings and opportunities for prognostic and therapeutic innovation. Biol Psychiatry. (2020) 88:95–102. 10.1016/j.biopsych.2019.09.004 31668548 PMC7075720

[13] Flippo KH Strack S. An emerging role for mitochondrial dynamics in schizophrenia. Schizophr Res. (2017) 187:26–32. 10.1016/j.schres.2017.05.003 28526279 PMC5646380

[14] Kim Y Vadodaria KC Lenkei Z Kato T Gage FH Marchetto MC. Mitochondria, metabolism, and redox mechanisms in psychiatric disorders. Antioxid Redox Signal. (2019) 31:275–317. 10.1089/ars.2018.7606 30585734 PMC6602118

[15] Eggan SM Hashimoto T Lewis DA. Reduced cortical cannabinoid 1 receptor messenger RNA and protein expression in schizophrenia. Arch Gen Psychiatry. (2008) 65:772–84. 10.1001/archpsyc.65.7.772 18606950 PMC2890225

[16] Volk DW Eggan SM Horti AG Wong DF Lewis DA. Reciprocal alterations in cortical cannabinoid receptor 1 binding relative to protein immunoreactivity and transcript levels in schizophrenia. Schizophr Res. (2014) 159:124–9. 10.1016/j.schres.2014.07.017 25107849 PMC4177350

[17] Bowen EFW Burgess JL Granger R Kleinman JE Rhodes CH. DLPFC transcriptome defines two molecular subtypes of schizophrenia. Transl Psychiatry. (2019) 9:147. 10.1038/s41398-019-0472-z 31073119 PMC6509343

[18] Bross M Hackett M Bernitsas E. Approved and emerging disease modifying therapies on neurodegeneration in multiple sclerosis. Int J Mol Sci. (2020) 21:4312. 10.3390/ijms21124312 32560364 PMC7348940

[19] Liu H Jin H Yue X Luo Z Liu C Rosenberg AJ. PET imaging study of S1PR1 expression in a rat model of multiple sclerosis. Mol Imaging Biol. (2016) 18:724–32. 10.1007/s11307-016-0944-y 26975859 PMC5297893

[20] Jin H Yang H Liu H Zhang Y Zhang X Rosenberg AJ. A promising carbon-11-labeled sphingosine-1-phosphate receptor 1-specific PET tracer for imaging vascular injury. J Nucl Cardiol. (2017) 24:558–70. 10.1007/s12350-015-0391-1 26843200

[21] Liu H Jin H Yue X Han J Baum P Abendschein DR. PET study of sphingosine-1-phosphate receptor 1 expression in response to vascular inflammation in a rat model of carotid injury. Mol Imaging. (2017) 16:1536012116689770. 10.1177/1536012116689770 28654378 PMC5470136

[22] Luo Z Gu J Dennett RC Gaehle GG Perlmutter JS Chen DL. Automated production of a sphingosine-1 phosphate receptor 1 (S1P1) PET radiopharmaceutical [(11)C]CS1P1 for human use. Appl Radiat Isot. (2019) 152:30–6. 10.1016/j.apradiso.2019.06.029 31280104 PMC6708718

[23] Liu H Luo Z Gu J Jiang H Joshi S Shoghi KI. *In vivo* characterization of four (18)F-labeled S1PR1 tracers for neuroinflammation. Mol Imaging Biol. (2020) 22:1362–9. 10.1007/s11307-020-01514-8 32602083 PMC7679043

[24] Jiang H Joshi S Liu H Mansor S Qiu L Zhao H. *In vitro* and *in vivo* investigation of S1PR1 expression in the central nervous system using [(3)H]CS1P1 and [(11)C]CS1P1. ACS Chem Neurosci. (2021) 12:3733–44. 10.1021/acschemneuro.1c00492 34516079 PMC8605766

[25] Williams MA. Autoradiography and Immunocytochemistry: Practical Methods in Electron Microscopy. North Holland: Elsevier Biomedical Press (1979). p. 6.

[26] Griem-Krey N Klein AB Herth M Wellendorph P. Autoradiography as a simple and powerful method for visualization and characterization of pharmacological targets. JoVE. (2019) 145:e58879. 10.3791/58879 30933077

[27] Nishimura H Akiyama T Irei I Hamazaki S Sadahira Y. Cellular localization of sphingosine-1-phosphate receptor 1 expression in the human central nervous system. J Histochem Cytochem. (2010) 58:847–56. 10.1369/jhc.2010.956409 20566754 PMC2924800

[28] Esaki K Balan S Iwayama Y Shimamoto-Mitsuyama C Hirabayashi Y Dean B. Evidence for altered metabolism of sphingosine-1-phosphate in the corpus callosum of patients with schizophrenia. Schizophr Bull. (2020) 46:1172–81. 10.1093/schbul/sbaa052 32346731 PMC7505171

[29] Singh SK Kordula T Spiegel S. Neuronal contact upregulates astrocytic sphingosine-1-phosphate receptor 1 to coordinate astrocyte-neuron cross communication. Glia. (2022) 70:712–27. 10.1002/glia.24135 34958493 PMC9219554

[30] Najjar S Pearlman DM. Neuroinflammation and white matter pathology in schizophrenia: systematic review. Schizophr Res. (2015) 161:102–12. 10.1016/j.schres.2014.04.041 24948485

[31] Comer AL Carrier M Tremblay ME Cruz-Martin A. The inflamed brain in schizophrenia: the convergence of genetic and environmental risk factors that lead to uncontrolled neuroinflammation. Front Cell Neurosci. (2020) 14:274. 10.3389/fncel.2020.00274 33061891 PMC7518314

[32] Potvin S Stip E Sepehry AA Gendron A Bah R Kouassi E. Inflammatory cytokine alterations in schizophrenia: a systematic quantitative review. Biol Psychiatry. (2008) 63:801–8. 10.1016/j.biopsych.2007.09.024 18005941

[33] Upthegrove R Manzanares-Teson N Barnes NM. Cytokine function in medication-naive first episode psychosis: a systematic review and meta-analysis. Schizophr Res. (2014) 155:101–8. 10.1016/j.schres.2014.03.005 24704219

[34] Kraguljac NV Mcdonald WM Widge AS Rodriguez CI Tohen M Nemeroff CB. Neuroimaging biomarkers in schizophrenia. Am J Psychiatry. (2021) 178:509–21. 10.1176/appi.ajp.2020.20030340 33397140 PMC8222104

[35] Kenk M Selvanathan T Rao N Suridjan I Rusjan P Remington G. Imaging neuroinflammation in gray and white matter in schizophrenia: an *in-vivo* PET study with [18F]-FEPPA. Schizophr Bull. (2015) 41:85–93. 10.1093/schbul/sbu157 25385788 PMC4266311

[36] Marques TR Ashok AH Pillinger T Veronese M Turkheimer FE Dazzan P. Neuroinflammation in schizophrenia: meta-analysis of *in vivo* microglial imaging studies. Psychol Med. (2019) 49:2186–96. 10.1017/S0033291718003057 30355368 PMC6366560

[37] Meyer JH Cervenka S Kim M-J Kreisl WC Henter ID Innis RB. Neuroinflammation in psychiatric disorders: PET imaging and promising new targets. The Lancet Psychiatry. (2020) 7:1064–74. 10.1016/S2215-0366(20)30255-8 33098761 PMC7893630

[38] Sneeboer MaM Van Der Doef T Litjens M Psy NBB Melief J. Microglial activation in schizophrenia: is translocator 18kDa protein (TSPO) the right marker? Schizophr Res. (2020) 215:167–72. 10.1016/j.schres.2019.10.045 31699629

[39] Marciniak A Camp SM Garcia JGN Polt R. *In silico* docking studies of fingolimod and S1P1 agonists. Front Pharmacol. (2020) 11:247. 10.3389/fphar.2020.00247 32210822 PMC7076195

[40] Chand GB Dwyer DB Erus G Sotiras A Varol E Srinivasan D. Two distinct neuroanatomical subtypes of schizophrenia revealed using machine learning. Brain. (2020) 143:1027–38. 10.1093/brain/awaa025 32103250 PMC7089665

